# Peutz-Jeghers Syndrome With Malignant Transformation in a Hamartomatous Rectal Polyp: A Case Report

**DOI:** 10.7759/cureus.97533

**Published:** 2025-11-23

**Authors:** Omar Bahlaoui, Anass Nadi, Afafe Taiymi, Wafaa Khannoussi, Imane Ben El Brhdadi

**Affiliations:** 1 Gastroenterology and Hepatology, Mohammed VI University of Health Sciences, Casablanca, MAR; 2 Gastroenterology and Hepatology, Mohammed VI International University Hospital, Bouskoura, MAR

**Keywords:** carcinoma, dysplasia, hamartomatous polyp, peutz-jeghers syndrome, rectum

## Abstract

Peutz-Jeghers syndrome (PJS) is a rare hereditary condition characterized by mucocutaneous pigmentation and hamartomatous gastrointestinal polyps. We describe the case of a 14-year-old girl who presented with rectal bleeding, in whom endoscopic evaluation revealed multiple hamartomatous polyps, including two large rectal lesions. Histopathological analysis showed areas of high-grade dysplasia and in situ carcinoma developing within a hamartomatous polyp. Although this syndrome is generally benign, affected individuals have a lifelong predisposition to gastrointestinal and extra-digestive malignancies. Malignant transformation during adolescence is exceptionally uncommon, highlighting the importance of early diagnosis and structured surveillance to reduce cancer risk.

## Introduction

Peutz-Jeghers syndrome (PJS) is an autosomal dominant inherited disease with variable expressivity. It is a rare disease, with a frequency estimated in the general population between 1/100,000 and 1/200,000 births [1]. Diagnosis is often made in the second decade of life, with a median age of 11 years, without sex or race predominance [1].

It is characterized by the presence of noncancerous growths called hamartomatous polyps in the digestive tract, classically associated with cutaneous-mucous pigmentation. It is caused by a germline mutation in the tumor suppressor gene STK11 located on the short arm of chromosome 19 [2].

This syndrome was first described in 1921 by the Dutchman Peutz and was detailed 28 years later by the American Jeghers [3]. Diagnostic criteria were established by the WHO in 2000 and then revised in 2007 by a European expert consensus [4].

The hamartomatous polyps of PJS vary in size and can cause obstruction or bleeding. In the digestive tract, they are mainly located in the small intestine (70-90%), colon (50%), and rectum (25%), but can also be found in the gallbladder, urinary tract, bronchi, and nasal orifices [4].

Beyond these mechanical complications, PJS is associated with a substantially increased lifetime risk of gastrointestinal and extra-intestinal malignancies. Several studies have reported cases of hamartomatous polyps undergoing malignant transformation through a proposed hamartoma-dysplasia-carcinoma sequence, most often in adult patients and usually later in life.

Malignant transformation within a hamartomatous polyp in childhood is exceptionally rare, and it is seldom the first manifestation of the disease. We report the case of a 14-year-old girl who presented with in situ carcinoma arising in an inaugural hamartomatous rectal polyp, revealing PJS.

## Case presentation

A 14-year-old female patient, the second of four children, with no significant medical history, presented to the emergency department with a 15-day history of recurrent, low-volume rectal bleeding.

**Figure 1:**
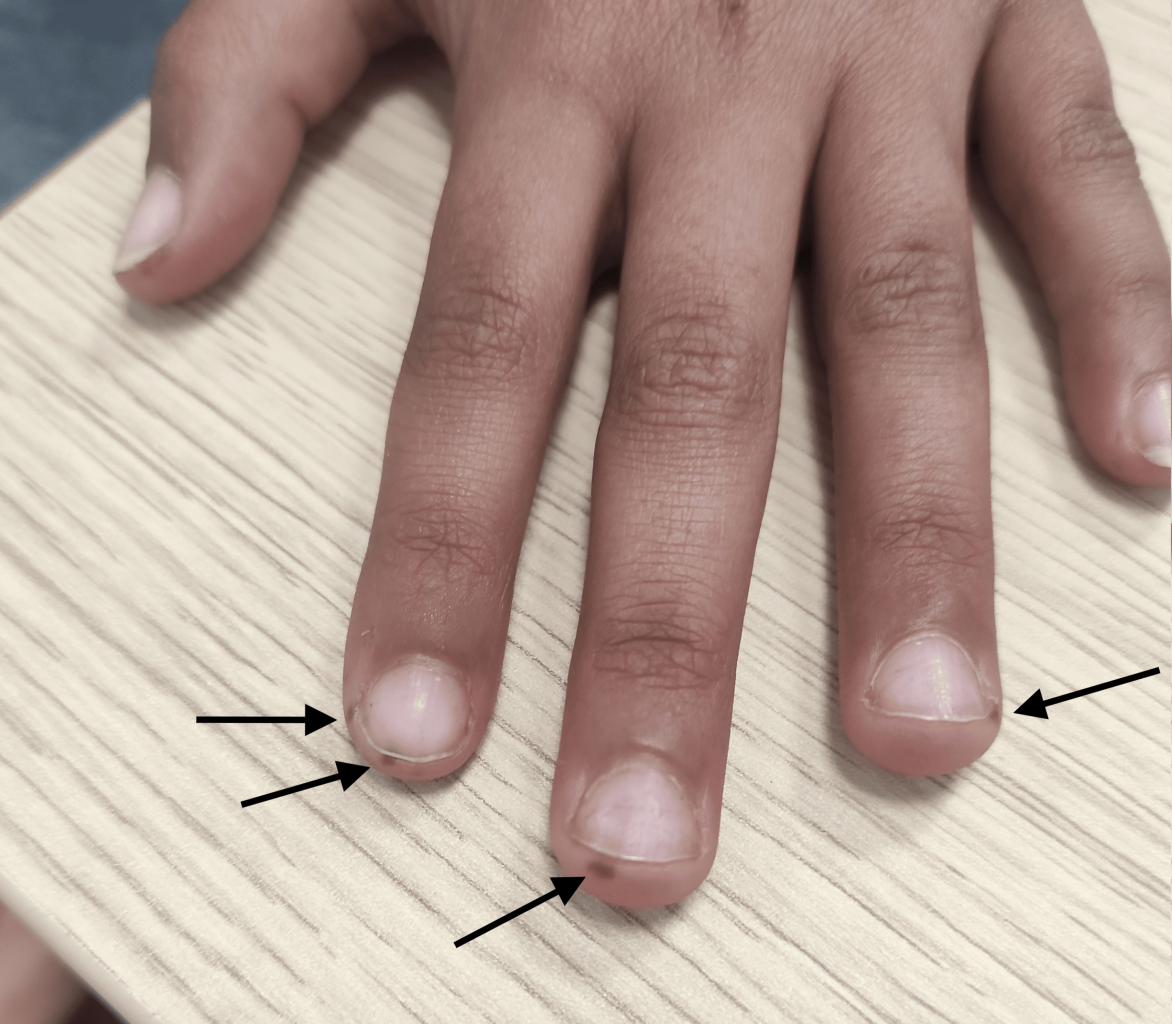
Mucocutaneous pigmentation on the fingertips in a patient with Peutz-Jeghers syndrome. Small, well-defined brown macules are visible on the fingers, characteristic of the mucocutaneous melanotic pigmentation typically seen in Peutz-Jeghers syndrome.

**Figure 2:**
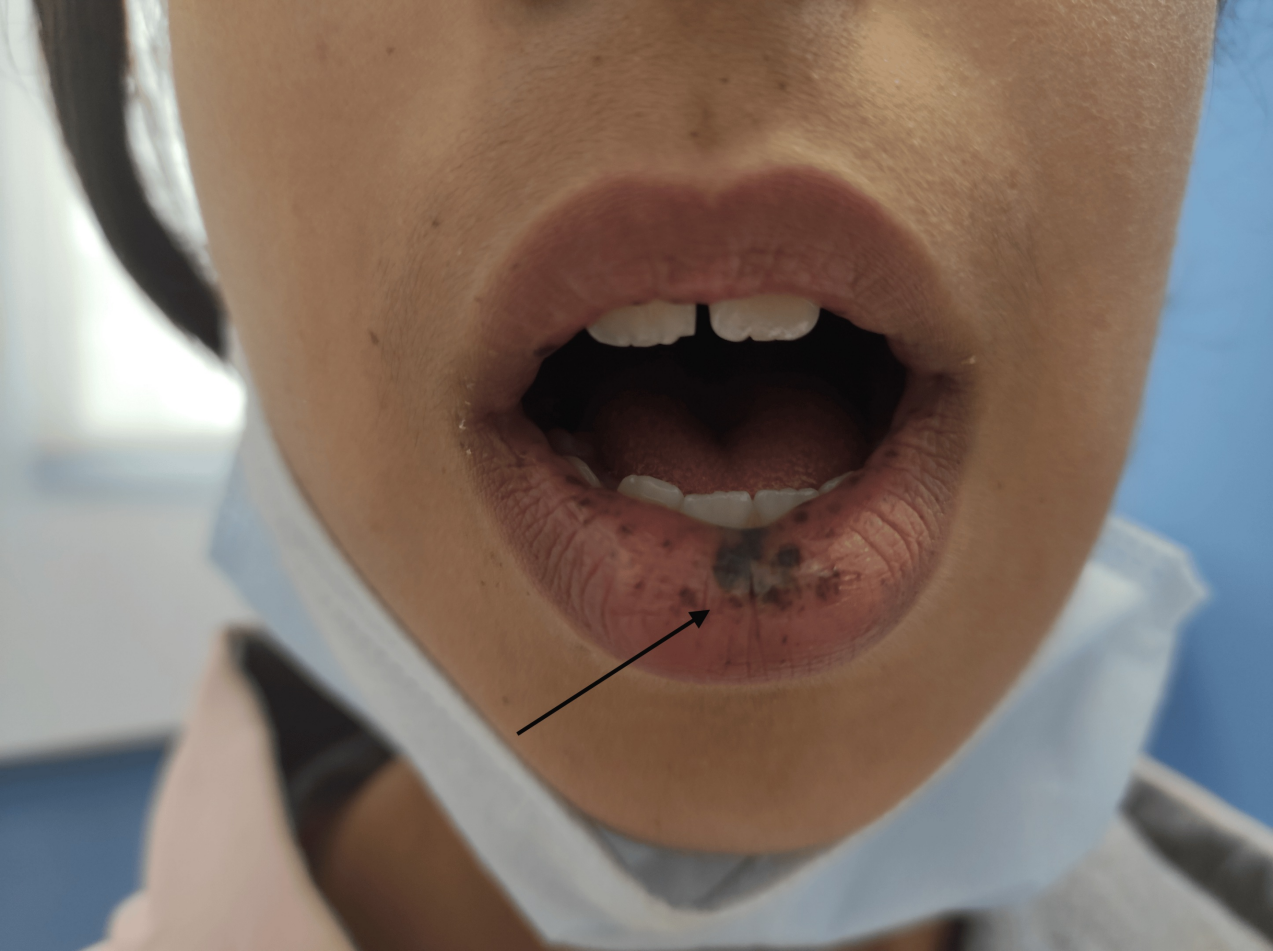
Mucocutaneous pigmentation of the lips in a patient with Peutz-Jeghers syndrome. Dark-brown to black macules are visible on the lower lip and perioral region, characteristic of the mucocutaneous melanotic pigmentation typically associated with Peutz-Jeghers syndrome.

Physical examination revealed a stable patient with normal hemodynamic and respiratory status. Skin examination revealed pigmented spots on the face, lips, and fingers of both hands (Figures 1, 2). Abdominal examination showed a soft abdomen, and rectal examination revealed a blood-stained glove finger. The rest of the physical examination was normal. 

**Figure 3:**
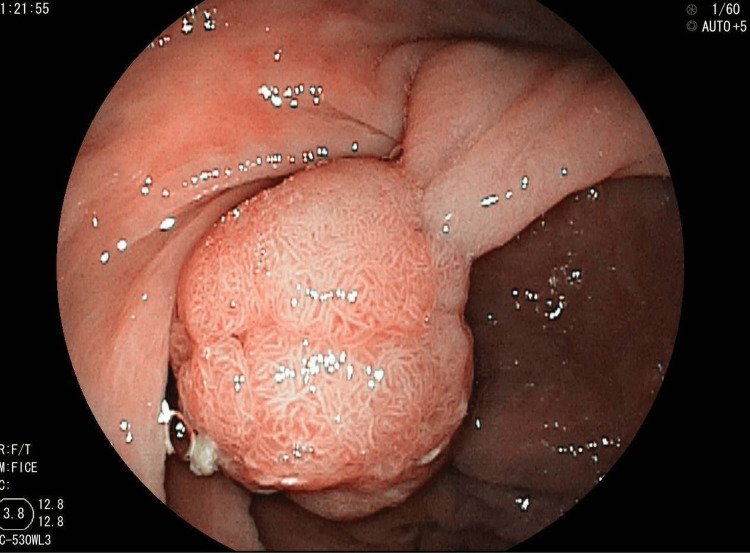
Endoscopic view of a large pedunculated rectal polyp in a patient with Peutz-Jeghers syndrome. Colonoscopy revealed a smooth, lobulated, pedunculated polyp in the rectum, consistent with a hamartomatous lesion characteristic of Peutz-Jeghers syndrome.

Colonoscopy revealed two large pedunculated rectal polyps, the first at 10 cm and the second at 12 cm from the anal margin, measuring 20 mm and 15 mm, respectively, and a small 5 mm polyp in the left colon (Figure 3). Esophagogastro duodenoscopy showed three small fundic polyps measuring 5 mm with erosive gastritis. Capsule endoscopy showed multiple diminutive polyps (<5 mm) scattered in the jejunum and ileum.

**Figure 4:**
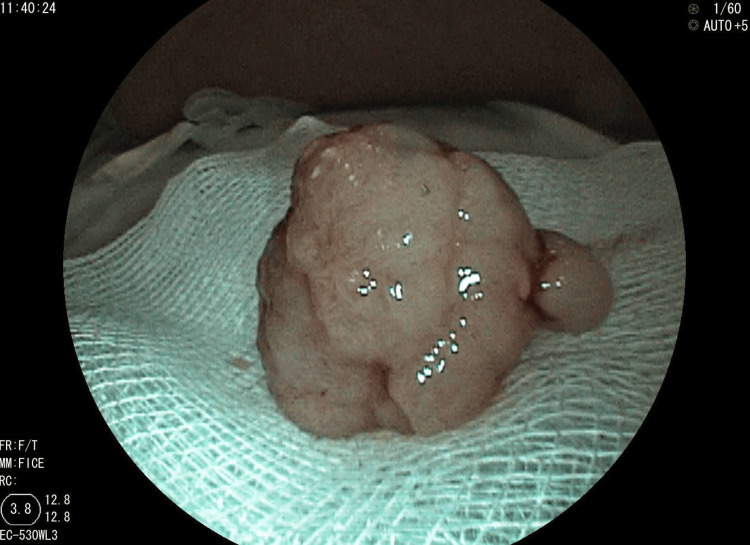
Resected pedunculated rectal polyp from a patient with Peutz-Jeghers syndrome. Gross view of the excised polyp obtained during endoscopic polypectomy, showing a lobulated and pedunculated hamartomatous lesion consistent with Peutz-Jeghers syndrome.

We performed polypectomy of the two large rectal polyps (Figure 4), while the other colonic and gastric polyps were resected by cold snare, and the small bowel polyps were left for surveillance.

Histological examination of the rectal polyp located 10 cm from the anal margin showed a hamartomatous aspect with stroma traversed by smooth muscle fibers associated with amphora-shaped crypts continuing on the surface by high-grade dysplastic lesions without adenomatous lesions.

Histological examination of the second rectal polyp located 12 cm from the anal margin showed carcinoma in situ without invasion of the submucosa with hamartomatous and high-grade dysplastic lesions.

Examination of the other colonic and gastric polyps was suggestive of hamartomatous lesions indicative of Peutz-Jeghers syndrome without dysplasia or carcinoma. A summary of all diagnostic investigations is provided in Table 1.

**Table 1:** Summary of diagnostic investigations.

Investigation	Findings
Laboratory tests	Hemoglobin 12 g/dL; normal ionogram; normal kidney function; normal coagulation studies
Colonoscopy	Two large pedunculated rectal polyps (20 mm and 15 mm) at 10 cm and 12 cm from the anal margin; one small 5 mm polyp in the left colon
Esophagogastroduodenoscopy	Three small fundic polyps (5 mm) associated with erosive gastritis
Capsule endoscopy	Multiple diminutive polyps (<5 mm) scattered in the jejunum and ileum
Histopathology	Rectal polyp at 10 cm: hamartomatous aspect with high-grade dysplasia; rectal polyp at 12 cm: carcinoma in situ without submucosal invasion; other colonic and gastric polyps: hamartomatous lesions without dysplasia or carcinoma
Computed tomography (CT) of the chest, abdomen, and pelvis	Normal
Tumor marker tests	Normal
Gynecological and thyroid examinations	Normal
Screening endoscopies of parents and siblings	No abnormalities detected

Histopathological analysis was performed in an external reference pathology laboratory. Due to institutional data-sharing restrictions, representative histopathology images could not be included in this report.

Tomography (CT) of the chest, abdomen, and pelvis and tumor marker tests were normal. Gynecological and thyroid examinations were normal. We performed screening digestive endoscopies on the parents and siblings, which revealed no abnormalities.

After endoscopic resection and completion of the staging workup, the patient was enrolled in a regular surveillance program, including periodic clinical review and scheduled endoscopic follow-up adapted to Peutz-Jeghers syndrome-related cancer risk, and she continues to be followed in our structure.

## Discussion

PJS is a rare autosomal dominant hereditary disease characterized by hamartomatous digestive polyposis associated with periorificial cutaneous and mucosal pigmentary lesions (lips, nose, anal and genital regions), with an increased risk of digestive and extra-digestive cancers. Diagnosis is often established during the second decade of life. Clinical manifestations are mainly due to complications of the polyps, such as intestinal invagination and digestive bleeding [4].

In our case, the diagnosis of PJS was established according to the WHO diagnostic criteria in a 14-year-old adolescent with rectal bleeding and no family history of digestive polyposis. Our clinical case reports a rare aspect of PJS, namely, malignant degeneration of a hamartomatous rectal polyp at a very young age, which is an extremely rare phenomenon in children and adolescents [5]. In a series of 2,500 polypectomies, dysplasia/atypia was observed in only 0.24% of cases [5].

It is widely accepted that there is a risk of cancer in PJS. However, this data is mostly based on single-center studies with small sample sizes and biases, making it difficult to draw conclusions. The seminal study by Jeghers et al. [3] reported digestive cancers associated with PJS, and since then, the number of reported cases has increased. However, the risk of digestive cancers in PJS is difficult to evaluate due to the rarity of the syndrome and the retrospective nature of the studies [6]. In the meta-analysis by Hearle et al. that included 419 patients with PJS, the risk of gastrointestinal cancer was estimated at 1% at 30 years, 9% at 40 years, 33% at 60 years, and increasing to 57% at 70 years [7].

Regarding the risk of colorectal cancer, it was estimated to be 3% at 30 years and 28% at 60 years in the study by Chen et al. [8], and 36% at 70 years in the study by Ishida et al. [9]. This risk of cancer is probably overestimated due to the retrospective nature and small sample sizes of the studies [6].

The second particularity of our clinical case is the histopathology of the resected polyp. Indeed, the pathological study revealed hamartomatous lesions associated with high-grade dysplasia and in situ carcinoma, suggesting an unusual carcinogenesis sequence of hamartoma-dysplasia-carcinoma. The carcinogenesis pathway in PJS is a highly controversial subject. The degeneration of hamartomatous polyps has sparked much debate and has been the subject of numerous controversies [10]. Other hypotheses of carcinogenesis on flat mucosa or adenomatous polyps are still plausible.

Our clinical case supports the hypothesis of the hamartoma-dysplasia-carcinoma pathway without excluding other hypotheses. This new concept of hamartoma-dysplasia-cancer may lead to new follow-up and treatment modalities [11].

## Conclusions

PJS is a rare genetic disorder that carries a substantial risk of digestive and extra-digestive cancers. Malignant degeneration of a hamartomatous polyp at a young age is an exceptionally rare event but should alert gastroenterologists, pediatricians, and pathologists to the possibility of early neoplastic transformation in this setting.

This case underlines the importance of early recognition of mucocutaneous pigmentation, prompt investigation of rectal bleeding, and the implementation of structured, lifelong surveillance within a multidisciplinary team for children and adolescents with PJS. While a single case cannot define management algorithms, it highlights the need for international collaborative efforts and large-scale studies to better quantify early cancer risk and to develop evidence-based follow-up protocols for this vulnerable population.
